# Retinal Diseases Caused by Mutations in Genes Not Specifically Associated with the Clinical Diagnosis

**DOI:** 10.1371/journal.pone.0165405

**Published:** 2016-10-27

**Authors:** Xia Wang, Yanming Feng, Jianli Li, Wei Zhang, Jing Wang, Richard A. Lewis, Lee-Jun Wong

**Affiliations:** 1 Department of Molecular and Human Genetics, Baylor College of Medicine, Houston, Texas, United States of America; 2 Baylor Genetics, Houston, Texas, United States of America; 3 Department of Ophthalmology, Baylor College of Medicine, Houston, Texas, United States of America; Centro de Investigacion Principe Felipe, SPAIN

## Abstract

**Purpose:**

When seeking a confirmed molecular diagnosis in the research setting, patients with one descriptive diagnosis of retinal disease could carry pathogenic variants in genes not specifically associated with that description. However, this event has not been evaluated systematically in clinical diagnostic laboratories that validate fully all target genes to minimize false negatives/positives.

**Methods:**

We performed targeted next-generation sequencing analysis on 207 ocular disease-related genes for 42 patients whose DNA had been tested negative for disease-specific panels of genes known to be associated with retinitis pigmentosa, Leber congenital amaurosis, or exudative vitreoretinopathy.

**Results:**

Pathogenic variants, including single nucleotide variations and copy number variations, were identified in 9 patients, including 6 with variants in syndromic retinal disease genes and 3 whose molecular diagnosis could not be distinguished easily from their submitted clinical diagnosis, accounting for 21% (9/42) of the unsolved cases.

**Conclusion:**

Our study underscores the clinical and genetic heterogeneity of retinal disorders and provides valuable reference to estimate the fraction of clinical samples whose retinal disorders could be explained by genes not specifically associated with the corresponding clinical diagnosis. Our data suggest that sequencing a larger set of retinal disorder related genes can increase the molecular diagnostic yield, especially for clinically hard-to-distinguish cases.

## Introduction

Inherited retinal diseases are a heterogeneous group of disorders that lead to retinal dysfunction and visual impairment. Retinitis pigmentosa (RP) is a group of progressive retinal dystrophies affecting about 1 in 3000 individuals [1,2]. RP causes night blindness and progressive loss of peripheral vision in early stages and loss of central vision later in life. Leber congenital amaurosis (LCA) represents a group of severe retinal disorders causing profound visual disability recognizable shortly after birth or within the first year of life. LCA affects about 1 in 50,000 people and is characterized by early onset visual impairment, nystagmus, and non or poorly recordable responses in the electroretinogram (ERG) [3]. Familial exudative vitreoretinopathy (FEVR) is a retinal disorder associated with defective retinal angiogenesis. FEVR is characterized by avascularity in the peripheral retina with variable clinical presentations, from no symptoms to early onset blindness [4]. To date, pathogenic variants in about 55, 19, and 5 genes are known to cause non-syndromic RP, LCA, and FEVR, respectively [5–7].

Targeted next-generation sequencing (NGS) has been used extensively for the molecular diagnosis of retinal diseases [8,9]. The diagnostic yields of targeted NGS panels range from 36% to 82% for RP 18% to 40% for LCA, and 49% for FEVR [6,10–14]. It has been reported that patients with a descriptive clinical diagnosis of retinal disease may carry pathogenic variants in genes not specifically associated with that diagnosis due to the substantive phenotypic overlap and genetic heterogeneity [6,15–17]. For example, apparently non-syndromic patients with retinitis pigmentosa may carry pathogenic variants in the Bardet-Biedl syndrome gene, *BBS1* [18]. Patients with severe visual impairments can have pathogenic variants in pattern dystrophy gene *PRPH2* [6]. Thus, tests focused on a specific group of genes for a particular clinical diagnosis may not detect variants in genes not typically associated with that condition. Despite a few reports in research settings, this phenomenon has not been evaluated systematically in clinical diagnostic laboratories that fully validate all target genes to minimize both false negatives and false positives [12].

Previously, our laboratory analyzed 98 RP, 13 LCA, and 12 FEVR samples by targeted capture NGS. A total of 207 ocular disease genes were captured and sequenced for each of these samples (S1 File). However, we focused the sequence analysis on 66 RP, 19 LCA, and 4 FEVR genes that have been clinically validated and are well known to be associated with the corresponding disorders. As a result, definitive molecular diagnoses were previously established in 73% (72/98) of RP, 46% (6/13) of LCA, and 25% (3/12) of FEVR cases, which are similar to previously published results mentioned above (S1 Table).

We hypothesized that a portion of the unsolved cases might be caused by pathogenic variants in other retinal disease genes not analyzed initially. Since the sequence data of 207 ocular disease-related genes are readily available, we analyzed the remaining genes of the 42 unsolved cases in this study. Our data underscore the clinical and genetic heterogeneity of retinal disorders and suggest that sequencing a larger set of related retinal disease genes can increase the molecular diagnostic yield.

## Materials and Methods

### Patient samples

A total of 42 DNA samples tested negative for pathogenic variants in the clinically validated 66 RP, 19 LCA, or 4 FEVR genes at CLIA-certified and CAP-accredited Baylor Miraca Genetics Laboratories (BMGL) were further analyzed as described below. The subsequent analyses were performed by protocols approved by Institutional Review Board for Human Subject Research of Baylor College of Medicine, and complied with the tenets of the Declaration of Helsinki. Patient information was de-identified prior to the analysis.

### Sequencing analyses and variant interpretation

Our targeted capture NGS approach has been described recently [12]. Briefly, a custom-designed DNA probe library was used to capture target exons and 20bp of the flanking intron regions of 207 ocular disease genes (S1 File). Indexed captured samples were pooled to be loaded onto each lane of the flow cells for sequencing on a HiSeq2000 (Illumina, Inc., San Diego, CA, USA) with 100 cycle single-end reads. Clinical validations were performed for 66 RP, 19 LCA, and 4 FEVR genes that are well-known to be associated with the corresponding disorders (https://www.bcm.edu/research/medical-genetics-labs/, test code 2190, 5090, 5250). Those regions with coverage <20X, usually GC rich or highly repetitive, were covered by PCR/Sanger sequencing. An average of 1000X per base sequence depth was achieved and 3–12 candidate variants were obtained per sample [12]. American College of Medical Genetics guidance was used for the interpretation of sequence variants [19]. Pathogenic variants were confirmed by Sanger sequencing.

### Copy number variation analysis

Analysis and detection of exonic CNVs were performed according to our recently published method [20]. Briefly, normalized coverage of each exon of a test sample was compared to the mean coverage of the same exon in the reference samples. The exons with possible CNVs were depicted automatically. The script for the detection of CNVs is deposited at https://sourceforge.net/projects/cnvanalysis. Candidate CNVs were confirmed by a custom-designed oligonucleotide CGH array [21].

## Results

### Summary of identified pathogenic variants

Pathogenic variants in other retinal disease genes not previously analyzed were identified in five RP, two LCA, and two FEVR cases, accounting for 19% (5/26), 29% (2/7), and 22% (2/9) of unsolved RP, LCA, and FEVR cases, respectively (Tables 1 and 2). Additionally, single heterozygous pathogenic variant in autosomal recessive disorders were identified in two RP patients (data not shown). All the reported variants were confirmed by Sanger sequencing. Taken together, variants in other retinal disease genes were identified in 21% of (9/42) unsolved patients (Table 1).

**Table 1:** Summary of cases in this study.

Disease	Total initially unsolved cases	Solved by other retinal disease genes
RP	26	5 (19%)
LCA	7	2 (29%)
FEVR	9	2 (22%)
Total	42	9 (21%)

**Table 2:** Variants identified in genes not specifically associated with the corresponding disease.

Patient	Gender	Age (yrs)	Test Referred	Gene	Allele1	Allele2	Clinical features	Familial study
**Autosomal Recessive**								
1	M	10	RP	*BBS10*	c.1677C>A (p.Y559*)[22]	c.9_15delinsGC (p.S3Rfs*91)	Rod and cone dystrophy, horseshoe kidney, ureterocele.	
2	F	25	RP	*BBS1*	c.1169T>G(p.M390R)[23]	c.1645G>T(p.E549*)[23]	Retinitis pigmentosa	
3#	M	17	RP	*BBS1*	c.1169T>G(p.M390R)[23]	c.1169T>G(p.M390R)[23]	Rod and cone dystrophy, (excision of) an extra digit	Both parents are heterozygous for p.M390R
4	M	9	LCA	*ALMS1*	c.2816T>A (p.L939*)	c.8776C>T (p.R2926*)	Blindness, hearing loss, severe mental retardation	
5	F	8	LCA	*NPHP1*	c.625-2A>G	Whole gene deletion	Infantile nystagmus, poor vision from birth, non-recordable ERG	
6	M	45	RP	*DFNB31*	c.409dupG (p. E137Gfs*42)	c.409dupG (p. E137Gfs*42)	Retinitis pigmentosa, hearing loss	Affected sibling is homozygous for p.E137fs
**Autosomal Dominant**								
7	F	33	RP	*GUCA1A*	c.341C>T (p.T114I)[24]		Retinal dystrophy	
8	M	1	FEVR	*RIMS1*	c.3399-2delA		Bilateral retinal detachment, cataracts, leukocoria, possible hearing loss, delayed milestones	
**X Linked**								
9	M	13	FEVR	*RS1*	c.214G>A (p.E72K)[25]		Tractional retinal detachment, vitreous hemorrhage, retinal dragging, peripheral avascular retinas	

^#^: This patient has been previously reported [12].

### Patients with variants in syndromic retinal disease genes

**Fig 1:**
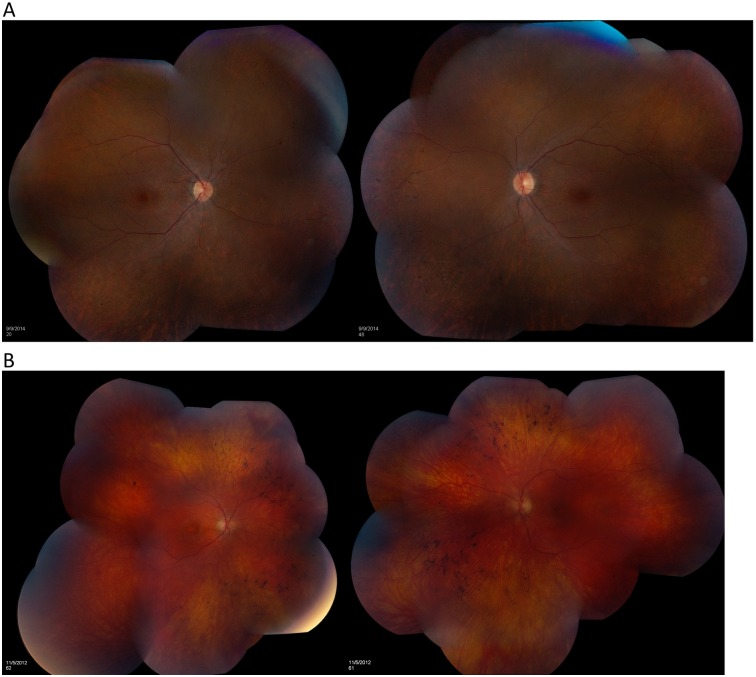
Retina features of patient 1 and 3. The retinal examination of patient 1 showed moderate diffuse pallor of each optic disc, moderate vascular attenuation, the dusky depigmentation of the retinal periphery, and small flecks of pigment migration into the retina, especially in the nasal hemispheres, all evidence of a widespread rod and cone dystrophy. (B) The retinal examination of patient 3 revealed slight diffuse pallor of each optic nerve, moderate attenuation of the retinal vasculature, and diffuse perimacular depigmentation with bone spicule pigment migration into the retina, especially in the nasal hemispheres, all evidence of a widespread rod and cone dystrophy.

**Fig 2:**
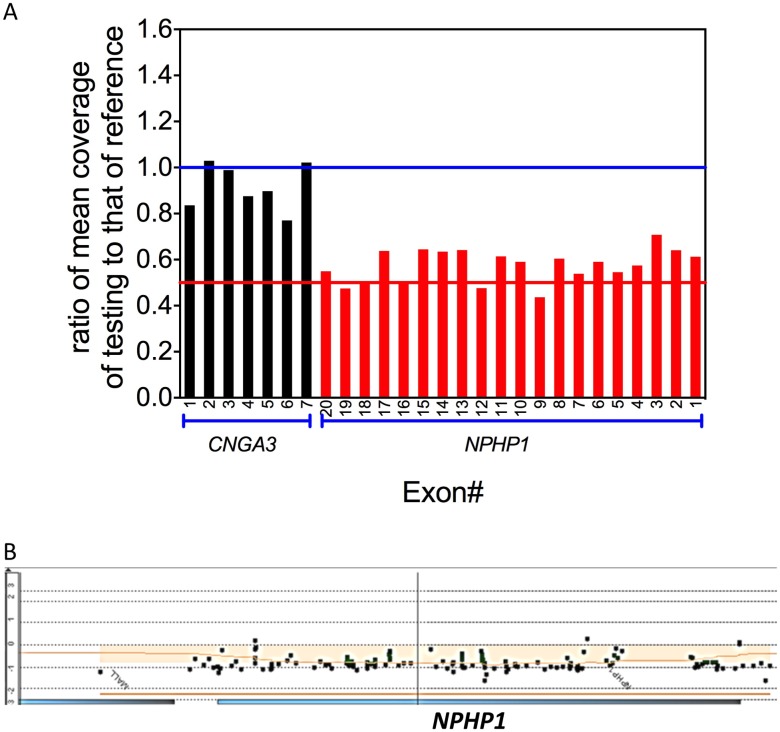
Detection of heterozygous *NPHP1* whole gene deletion in patient 5. (A) The ratio of normalized mean NGS coverage of individual coding exon of *CNGA3*, a gene on the same chromosome with *NPHP1*, and *NPHP1*, to that of the reference was plotted against the exon number. The normalization NGS coverage depth ratios of all exons of *NPHP1* are about 0.5, indicating heterozygous deletion. (B) The aCGH confirmation of the heterozygous *NPHP1* whole gene deletion. Log2 ratios of most probes on *NPHP1* gene are -1, suggesting heterozygous whole gene deletion.

Six of these patients have variants in syndromic retinal disease genes. Syndromic features other than an isolated retinal dystrophy may be detected or may develop later in life than the time at which the patient is evaluated for visual impairment. Thus, the additional systemic features may not have evolved or may be overlooked at the time of the initial ophthalmologic evaluation. These constitutional features may also be less evident than expected. Patient 1 harbors a heterozygous reported nonsense change, c.1677C>A (p.Y559*), and a heterozygous novel frameshift indel, c.9_15delinsGC (p.S3Rfs*91), in *BBS10* gene. Defects in *BBS10* cause Bardet-Biedl syndrome 10 (BBS10) [MIM: 615987], an autosomal recessive ciliopathy characterized by retinitis pigmentosa, obesity, kidney dysfunction, polydactyly, obsessive-compulsive behavior, and hypogonadism [26]. This patient had widespread rod and cone dystrophy but did not have obesity, speech pathology, intellectual disability, polydactyly, or hypogonadism (Fig 1A). However, the family subsequently disclosed that he was born with a horseshoe kidney and had had surgical repair of an ureterocele, which has been observed in BBS [27]. Patient 2, a 25-years-old woman, was referred for molecular diagnosis of non-syndromic RP and was found to have a heterozygous well established pathogenic variant, c.1169T>G (p.M390R), and a heterozygous nonsense variant, c.1645G>T(p.E549*), in the *BBS1* gene. Defects in *BBS1* cause Bardet-Biedl syndrome 1 (BBS1) [MIM: 209900], an autosomal recessive and genetically heterogeneous ciliopathy characterized by retinitis pigmentosa, obesity, kidney dysfunction, polydactyly, behavioral dysfunction, and hypogonadism. It has also been previously described that *BBS1* mutations can result in a wide spectrum of phenotypes, including apparently nonsyndromic retinitis pigmentosa, if other clinical features are not carefully sought for [18]. Patient 3 carries a homozygous, well known pathogenic variant, c.1169T>G (p.M390R), in *BBS1* gene. This patient had widespread rod and cone dystrophy but did not have obesity, developmental delay, speech pathology, intellectual disability, or renal defects (Fig 1B). However, after revealing the results for *BBS1* mutation, the parents disclosed the previous excision of a small extra digit, consistent with polydactyly in BBS [12]. Patient 4 carries compound heterozygous novel nonsense pathogenic variants, c.2816T>A (p.L939*) and c.8776C>T (p.R2926*), in the *ALMS1* gene. Defects in *ALMS1* cause Alstrom syndrome [MIM: 203800], an autosomal recessive disorder characterized by progressive cone-rod dystrophy leading to blindness, sensorineural hearing loss, childhood obesity associated with hyperinsulinemia, developmental delay, and late onset type 2 diabetes mellitus. Subsequent clinical evaluation confirmed that this patient indeed had hearing loss and intellectual impairment in addition to the profound visual impairment that had initiated the request for molecular testing, consistent with the sequential appearance of other features of Alstrom syndrome. Patient 5 had infantile nystagmus, poor vision from birth, a non-recordable ERG, and thus was referred for genetic testing of LCA. We identified a heterozygous novel splice site pathogenic variant (apparently homozygous), c.625-2A>G, and a one copy whole gene deletion, in the *NPHP1* gene (Fig 2). The deletion was initially identified by NGS and was subsequently confirmed by aCGH. Defects in *NPHP1* can cause Joubert syndrome [MIM:609583], juvenile nephronophthisis [MIM:256100], and Senior-Loken syndrome [MIM:266900], which all have renal abnormalities. Because of the molecular results, the patient was referred for renal evaluation; subsequent renal ultrasound examination at age eight revealed slightly enlarged and echogenic kidneys with poor corticomedullary differentiation, consistent with nephronophthisis. Patient 6 was a 45-years old man with a history of RP and hearing loss. He was referred for the molecular diagnosis using our RP panel. A homozygous pathogenic novel variant, c.409dupG (p. E137Gfs*42), in the *DFNB31* gene was identified. Defects in the *DFNB31* gene are associated with autosomal recessive Usher Syndrome Type IID characterized by hearing loss and retinitis pigmentosa [MIM:611383], and autosomal recessive deafness 31 [MIM: 607084]. The patient’s clinical phenotype is consistent with the molecular diagnosis. Our results suggest that syndromic retinal disease genes may account for a substantial portion of the undiagnosed, apparently non-syndromic, retinal disorder cases (14%, 6/42).

### Patients with variants in non-syndromic retinal disease genes

The working clinical diagnoses of some of these patients were confounded by their ages when first evaluated by the ophthalmologist. For example, patient 7 was referred for RP testing but was found to have a heterozygous variant, c.341C>T (p.T114I), in the *GUCA1A* gene after our expanded analysis. This variant was reported previously in a single patient with cone dystrophy but has never been reported in public databases [24]. It is known that mutations in *GUCA1A* cause autosomal dominant cone dystrophy or cone-rod dystrophy (CRD) [MIM: 602093]. Since this patient was 32 years old at the time of diagnosis, the retinal dystrophy seems to have progressed to a late stage for a clear clinical discrimination between RP and cone dystrophy/CRD. It is also possible that RP may be a new phenotypic variability of this mutation. Similarly, patient 8 carries a heterozygous novel deletion, c.3399-2delA, in the *RIMS1* gene. This variant changes the acceptor splice site of exon 23 and is very likely to cause exon 23 skipping. While not validated for clinical use, the MaxEntScan and Human Splicing Finder algorithms predict this change to completely abolish the acceptor splice site [28,29]. Nonsense and missense changes in *RIMS1* have been reported in autosomal dominant RP and CRD [30,31]. Patient 8 had bilateral retinal detachment and cataracts, which were not mentioned in those reported patients with *RIMS1* mutations but can be associated with RP [32]. Since this patient was evaluated at 1 year of age, he may have been too young for a unique clinical diagnosis.

Definitive molecular diagnosis also reveals the wide clinical spectrum of non-syndromic retinal diseases. Patient 9 was referred for FEVR testing because he had typical FEVR features including tractional retinal detachment, vitreous hemorrhage, retinal dragging, and peripheral avascular retina. However, our analysis identified a well-known hemizygous pathogenic variant, c.214G>A (p.E72K), in the X-linked retinoschisis (XLRS) gene *RS1* [25]. XLRS is characterized by splitting of the neural retina (schisis). Schisis occurs in the inner retinal layer and is usually different from the retinal detachment in FEVR, which is the split between the neural retina and the retinal pigment epithelium [33]. It has been reported that some XLRS patients with *RS1* mutations had atypical fundus findings, including tractional retinal dragging, exudative detachment, and vitreous hemorrhage, all of which are consistent with the clinical presentation of patient 9 [34,35]. Therefore, our data demonstrate that molecular diagnosis can refine or modify the descriptive clinical diagnosis and subsequently change the counseling for associated features, other complications, and recurrence risks for both the patient and the family.

## Discussion

Our study suggests that a substantial portion of undiagnosed and apparently non-syndromic retinal dystrophy cases can be explained by pathogenic variants in genes not specifically associated with the corresponding clinical diagnosis. We evaluated this hypothesis systematically by analyzing sequence variants in 207 ocular disease-related genes and identified pathogenic variants in genes not specifically associated with the corresponding clinical diagnosis in 9 out of 42 cases that could not be explained by smaller set of disease-specific genes. These cases account for 19% (5/26) of RP, 29% (2/7) of LCA, and 22% (2/9) of FEVR cases in our unsolved patient cohort (S1 Table). Our analysis increases the overall diagnostic rate from 73% to 78% for RP. The increases in diagnostic rate for LCA and FEVR are much higher, but the overall solved rates remain much lower than that for RP. The increases may be underestimates, because in this study we only focused on defined pathogenic variants and excluded variants of unknown significance. Nevertheless, our results suggest that sequencing a larger set of retinal disorder-related genes can increase substantially the diagnostic yield and help to identify unexpected genotype-phenotype correlations.

Our study provides valuable reference to estimate the fraction of clinical samples whose retinal disorders may be explained by genes not specifically associated with the corresponding clinical diagnosis. Similar findings have been reported elsewhere [6,15–17,36–41]. Our approach has unique advantages. We ensured 100% coverage by fill in the low or no coverage regions such as ORF15 of *RPGR* gene, or regions with high GC content and/or homologous sequences, by Sanger sequencing of specifically amplified PCR products. In addition, the average coverage depth is consistently at ~1000X per base, that allows the detection of heterozygous exonic CNVs [20]. The CNV detection algorithm has been integrated into the routine analytical pipeline for clinical application that has been validated in parallel with exon targeted oligonucleotide array CGH [20]. These unique features of our panel-based NGS approach greatly improve clinical sensitivity. With 100% coverage and the ability to detect SNVs and CNVs simultaneously, a negative result from deep NGS panel analysis suggests that the disease-causing variants are unlikely in the target regions, and other options such as WES may be considered. WES has been used for the molecular diagnosis of retinal disorders, however, it does not ensure 100% coverage and is not validated clinically for CNVs [42–45]. Thus, in our experience, if a sample with isolated retinal disorder is negative for all candidate genes in the targeted panel, the yield of additional WES analysis is minimal. Indeed, four of the samples in this study also had clinical WES in our laboratory, and no additional reportable variants were identified.

Early identification of mutations in syndromic retinal disease genes of apparently non-syndromic and isolated retinal disease patients could lead to timely and pre-planned management before acute presentation of more serious features of the complete syndrome [16,46]. Among the nine patients with variants in genes not specifically associated with the corresponding clinical diagnosis, six carry variants in syndromic retinal disease genes. It has been reported that pathogenic variants in syndromic retinal disease genes can lead to wide spectrum of phenotypes, from non-syndromic retinal dystrophy to full syndromes [6,16,18]. In addition, it is not unusual that ophthalmologists tend to attend more to the ocular findings of the patient, while extra-ocular syndromic features were either unsought by the enquiring physician, unreported by the patient or family, or not yet developed at the time of eye evaluation. Therefore, it is important for physicians to gather clinical data comprehensively and be aware of the phenotypic overlapping among many retinal disorders. The remaining three patients carry variants in other non-syndromic retinal disease genes. Many factors, such as age at evaluation, wide variance in the phenotypic spectrum of diseases, genetic modifiers, and environmental exposures, may confound the incisive distinction of subtle differences between two clinically similar retinal phenotypes. In all these situations, sequencing a larger set of related retinal disease genes can help to capture variants in unexpected genes, increase the molecular diagnostic rate, reach a definitive clinical diagnosis, and lead to accurate prognosis and improved management of the patient.

Other genetic and technical factors may account for the remaining molecular etiology of retinal diseases. First, highly GC-rich, highly repetitive and/or homologous regions could not be captured, sequenced, and aligned unambiguously by targeted capture NGS [47,48]. For example, the open reading frame 15 (ORF15) of *RPGR* gene, which is a RP mutational hotspot, contains a ~300 bp highly repetitive region that cannot be unambiguously analyzed by conventional capture NGS [12,49,50]. Long-range PCR followed by NGS may be used to identify variants in these regions. Second, nucleotide changes not at the canonical splice site, or changes in regulatory regions such as promoter, or 3’ and 5’ untranslated regions, may be disease-causing. For example, we have added specific probes to capture the frequent intronic pathogenic variant c.2991+1655A>G in *CEP290* in our panel [51]. Third, exonic deletion/duplications have been shown to cause retinal diseases [52–54]. We have developed recently a method to detect exonic CNVs efficiently with capture based NGS data [20]. Here, we successfully identified a one copy whole gene deletion of *NPHP1* in patient 5 (Fig 2). Our data suggest that exonic CNV analysis should be included in the NGS panel-based clinical testing of retinal diseases to increase the diagnostic yield. Lastly, novel disease genes, yet to be identified, may account for other unsolved retinal disease cases. To identify novel disease genes, whole exome sequencing or whole genome sequencing may be considered.

## Supporting Information

S1 File The genes and transcripts included in the capture design. (TXT)

S1 Table Summary of cases in initial analysis and in this study. (DOCX)

## References

[1] Haim M. Epidemiology of retinitis pigmentosa in Denmark. Acta Ophthalmol Scand Suppl. 2002; 1–34. 11921605 10.1046/j.1395-3907.2002.00001.x

[2] Hartong DT, Berson EL, Dryja TP. Retinitis pigmentosa. Lancet Lond Engl. 2006;368: 1795–1809. 10.1016/S0140-6736(06)69740-7 17113430

[3] den Hollander AI, Roepman R, Koenekoop RK, Cremers FPM. Leber congenital amaurosis: genes, proteins and disease mechanisms. Prog Retin Eye Res. 2008;27: 391–419. 10.1016/j.preteyeres.2008.05.003 18632300

[4] Poulter JA, Ali M, Gilmour DF, Rice A, Kondo H, Hayashi K, et al Mutations in TSPAN12 cause autosomal-dominant familial exudative vitreoretinopathy. Am J Hum Genet. 2010;86: 248–253. 10.1016/j.ajhg.2010.01.012 20159112 PMC2820188

[5] Zhao L, Wang F, Wang H, Li Y, Alexander S, Wang K, et al Next-generation sequencing-based molecular diagnosis of 82 retinitis pigmentosa probands from Northern Ireland. Hum Genet. 2015;134: 217–230. 10.1007/s00439-014-1512-7 25472526 PMC4347882

[6] Wang X, Wang H, Sun V, Tuan H-F, Keser V, Wang K, et al Comprehensive molecular diagnosis of 179 Leber congenital amaurosis and juvenile retinitis pigmentosa patients by targeted next generation sequencing. J Med Genet. 2013;50: 674–688. 10.1136/jmedgenet-2013-101558 23847139 PMC3932025

[7] Gilmour DF. Familial exudative vitreoretinopathy and related retinopathies. Eye Lond Engl. 2015;29: 1–14. 10.1038/eye.2014.70 25323851 PMC4289842

[8] Oishi M, Oishi A, Gotoh N, Ogino K, Higasa K, Iida K, et al Comprehensive molecular diagnosis of a large cohort of Japanese retinitis pigmentosa and Usher syndrome patients by next-generation sequencing. Invest Ophthalmol Vis Sci. 2014;55: 7369–7375. 10.1167/iovs.14-15458 25324289

[9] Seong M-W, Seo SH, Yu YS, Hwang J-M, Cho SI, Ra EK, et al Diagnostic application of an extensive gene panel for leber congenital amaurosis with severe genetic heterogeneity. J Mol Diagn JMD. 2015;17: 100–105. 10.1016/j.jmoldx.2014.09.003 25445212

[10] Simpson DA, Clark GR, Alexander S, Silvestri G, Willoughby CE. Molecular diagnosis for heterogeneous genetic diseases with targeted high-throughput DNA sequencing applied to retinitis pigmentosa. J Med Genet. 2011;48: 145–151. 10.1136/jmg.2010.083568 21147909

[11] Neveling K, Collin RWJ, Gilissen C, van Huet RAC, Visser L, Kwint MP, et al Next-generation genetic testing for retinitis pigmentosa. Hum Mutat. 2012;33: 963–972. 10.1002/humu.22045 22334370 PMC3490376

[12] Wang J, Zhang VW, Feng Y, Tian X, Li F-Y, Truong C, et al Dependable and efficient clinical utility of target capture-based deep sequencing in molecular diagnosis of retinitis pigmentosa. Invest Ophthalmol Vis Sci. 2014;55: 6213–6223. 10.1167/iovs.14-14936 25097241

[13] Coppieters F, De Wilde B, Lefever S, De Meester E, De Rocker N, Van Cauwenbergh C, et al Massively parallel sequencing for early molecular diagnosis in Leber congenital amaurosis. Genet Med Off J Am Coll Med Genet. 2012;14: 576–585. 10.1038/gim.2011.51 22261762

[14] Salvo J, Lyubasyuk V, Xu M, Wang H, Wang F, Nguyen D, et al Next-generation sequencing and novel variant determination in a cohort of 92 familial exudative vitreoretinopathy patients. Invest Ophthalmol Vis Sci. 2015;56: 1937–1946. 10.1167/iovs.14-16065 25711638 PMC4365990

[15] Liu X, Xiao J, Huang H, Guan L, Zhao K, Xu Q, et al Molecular Genetic Testing in Clinical Diagnostic Assessments That Demonstrate Correlations in Patients With Autosomal Recessive Inherited Retinal Dystrophy. JAMA Ophthalmol. 2015; 10.1001/jamaophthalmol.2014.5831 25611614

[16] Werdich XQ, Place EM, Pierce EA. Systemic diseases associated with retinal dystrophies. Semin Ophthalmol. 2014;29: 319–328. 10.3109/08820538.2014.959202 25325857

[17] Xu Y, Guan L, Xiao X, Zhang J, Li S, Jiang H, et al Mutation analysis in 129 genes associated with other forms of retinal dystrophy in 157 families with retinitis pigmentosa based on exome sequencing. Mol Vis. 2015;21: 477–486. 25999675 PMC4415588

[18] Estrada-Cuzcano A, Koenekoop RK, Senechal A, De Baere EBW, de Ravel T, Banfi S, et al BBS1 mutations in a wide spectrum of phenotypes ranging from nonsyndromic retinitis pigmentosa to Bardet-Biedl syndrome. Arch Ophthalmol Chic Ill 1960. 2012;130: 1425–1432. 10.1001/archophthalmol.2012.2434 23143442

[19] Richards CS, Bale S, Bellissimo DB, Das S, Grody WW, Hegde MR, et al ACMG recommendations for standards for interpretation and reporting of sequence variations: Revisions 2007. Genet Med Off J Am Coll Med Genet. 2008;10: 294–300. 10.1097/GIM.0b013e31816b5cae 18414213

[20] Feng Y, Chen D, Wang G-L, Zhang VW, Wong L-JC. Improved molecular diagnosis by the detection of exonic deletions with target gene capture and deep sequencing. Genet Med Off J Am Coll Med Genet. 2015;17: 99–107. 10.1038/gim.2014.80 25032985 PMC4338802

[21] Wang J, Zhan H, Li F-Y, Pursley AN, Schmitt ES, Wong L-J. Targeted array CGH as a valuable molecular diagnostic approach: experience in the diagnosis of mitochondrial and metabolic disorders. Mol Genet Metab. 2012;106: 221–230. 10.1016/j.ymgme.2012.03.005 22494545

[22] Billingsley G, Bin J, Fieggen KJ, Duncan JL, Gerth C, Ogata K, et al Mutations in chaperonin-like BBS genes are a major contributor to disease development in a multiethnic Bardet-Biedl syndrome patient population. J Med Genet. 2010;47: 453–463. 10.1136/jmg.2009.073205 20472660

[23] Mykytyn K, Nishimura DY, Searby CC, Shastri M, Yen H, Beck JS, et al Identification of the gene (BBS1) most commonly involved in Bardet-Biedl syndrome, a complex human obesity syndrome. Nat Genet. 2002;31: 435–438. 10.1038/ng935 12118255

[24] Nishiguchi KM, Sokal I, Yang L, Roychowdhury N, Palczewski K, Berson EL, et al A novel mutation (I143NT) in guanylate cyclase-activating protein 1 (GCAP1) associated with autosomal dominant cone degeneration. Invest Ophthalmol Vis Sci. 2004;45: 3863–3870. 10.1167/iovs.04-0590 15505030 PMC1475955

[25] The Retinoschisis Consortium. Functional implications of the spectrum of mutations found in 234 cases with X-linked juvenile retinoschisis. The Retinoschisis Consortium. Hum Mol Genet. 1998;7: 1185–1192. 9618178 10.1093/hmg/7.7.1185

[26] Beales PL, Elcioglu N, Woolf AS, Parker D, Flinter FA. New criteria for improved diagnosis of Bardet-Biedl syndrome: results of a population survey. J Med Genet. 1999;36: 437–446. 10874630 PMC1734378

[27] Imhoff O, Marion V, Stoetzel C, Durand M, Holder M, Sigaudy S, et al Bardet-Biedl syndrome: a study of the renal and cardiovascular phenotypes in a French cohort. Clin J Am Soc Nephrol CJASN. 2011;6: 22–29. 10.2215/CJN.03320410 20876674 PMC3022245

[28] Yeo G, Burge CB. Maximum entropy modeling of short sequence motifs with applications to RNA splicing signals. J Comput Biol J Comput Mol Cell Biol. 2004;11: 377–394. 10.1089/1066527041410418 15285897

[29] Desmet F-O, Hamroun D, Lalande M, Collod-Béroud G, Claustres M, Béroud C. Human Splicing Finder: an online bioinformatics tool to predict splicing signals. Nucleic Acids Res. 2009;37: e67 10.1093/nar/gkp215 19339519 PMC2685110

[30] Glöckle N, Kohl S, Mohr J, Scheurenbrand T, Sprecher A, Weisschuh N, et al Panel-based next generation sequencing as a reliable and efficient technique to detect mutations in unselected patients with retinal dystrophies. Eur J Hum Genet EJHG. 2014;22: 99–104. 10.1038/ejhg.2013.72 23591405 PMC3865404

[31] Johnson S, Halford S, Morris AG, Patel RJ, Wilkie SE, Hardcastle AJ, et al Genomic organisation and alternative splicing of human RIM1, a gene implicated in autosomal dominant cone-rod dystrophy (CORD7). Genomics. 2003;81: 304–314. 12659814 10.1016/s0888-7543(03)00010-7

[32] Jackson H, Garway-Heath D, Rosen P, Bird AC, Tuft SJ. Outcome of cataract surgery in patients with retinitis pigmentosa. Br J Ophthalmol. 2001;85: 936–938. 10.1136/bjo.85.8.936 11466249 PMC1724090

[33] Sikkink SK, Biswas S, Parry NRA, Stanga PE, Trump D. X-linked retinoschisis: an update. J Med Genet. 2007;44: 225–232. 10.1136/jmg.2006.047340 17172462 PMC2598044

[34] Shukla D, Rajendran A, Gibbs D, Suganthalakshmi B, Zhang K, Sundaresan P. Unusual manifestations of x-linked retinoschisis: clinical profile and diagnostic evaluation. Am J Ophthalmol. 2007;144: 419–423. 10.1016/j.ajo.2007.05.016 17631851

[35] Greven CM, Moreno RJ, Tasman W. Unusual manifestations of X-linked retinoschisis. Trans Am Ophthalmol Soc. 1990;88: 211–225; discussion 226–228. 2095022 PMC1298587

[36] Perez-Carro R, Corton M, Sánchez-Navarro I, Zurita O, Sanchez-Bolivar N, Sánchez-Alcudia R, et al Panel-based NGS Reveals Novel Pathogenic Mutations in Autosomal Recessive Retinitis Pigmentosa. Sci Rep. 2016;6: 19531 10.1038/srep19531 26806561 PMC4726392

[37] Weisschuh N, Mayer AK, Strom TM, Kohl S, Glöckle N, Schubach M, et al Mutation Detection in Patients with Retinal Dystrophies Using Targeted Next Generation Sequencing. PLoS ONE. 2016;11: e0145951 10.1371/journal.pone.0145951 26766544 PMC4713063

[38] Bravo-Gil N, Méndez-Vidal C, Romero-Pérez L, González-del Pozo M, Rodríguez-de la Rúa E, Dopazo J, et al Improving the management of Inherited Retinal Dystrophies by targeted sequencing of a population-specific gene panel. Sci Rep. 2016;6: 23910 10.1038/srep23910 27032803 PMC4817143

[39] Consugar MB, Navarro-Gomez D, Place EM, Bujakowska KM, Sousa ME, Fonseca-Kelly ZD, et al Panel-based genetic diagnostic testing for inherited eye diseases is highly accurate and reproducible, and more sensitive for variant detection, than exome sequencing. Genet Med. 2015;17: 253–261. 10.1038/gim.2014.172 25412400 PMC4572572

[40] Chiang JP-W, Lamey T, McLaren T, Thompson JA, Montgomery H, De Roach J. Progress and prospects of next-generation sequencing testing for inherited retinal dystrophy. Expert Rev Mol Diagn. 2015;15: 1269–1275. 10.1586/14737159.2015.1081057 26394700 PMC4659341

[41] Daiger SP, Bowne SJ, Sullivan LS, Blanton SH, Weinstock GM, Koboldt DC, et al Application of next-generation sequencing to identify genes and mutations causing autosomal dominant retinitis pigmentosa (adRP). Adv Exp Med Biol. 2014;801: 123–129. 10.1007/978-1-4614-3209-8_16 24664689 PMC4121110

[42] Xu Y, Guan L, Shen T, Zhang J, Xiao X, Jiang H, et al Mutations of 60 known causative genes in 157 families with retinitis pigmentosa based on exome sequencing. Hum Genet. 2014;133: 1255–1271. 10.1007/s00439-014-1460-2 24938718

[43] Xu Y, Guan L, Xiao X, Zhang J, Li S, Jiang H, et al Mutation analysis in 129 genes associated with other forms of retinal dystrophy in 157 families with retinitis pigmentosa based on exome sequencing. Mol Vis. 2015;21: 477–486. 25999675 PMC4415588

[44] González-del Pozo M, Méndez-Vidal C, Bravo-Gil N, Vela-Boza A, Dopazo J, Borrego S, et al Exome sequencing reveals novel and recurrent mutations with clinical significance in inherited retinal dystrophies. PloS One. 2014;9: e116176 10.1371/journal.pone.0116176 25544989 PMC4278866

[45] Corton M, Nishiguchi KM, Avila-Fernández A, Nikopoulos K, Riveiro-Alvarez R, Tatu SD, et al Exome sequencing of index patients with retinal dystrophies as a tool for molecular diagnosis. PloS One. 2013;8: e65574 10.1371/journal.pone.0065574 23940504 PMC3683009

[46] Ellingford JM, Sergouniotis PI, Lennon R, Bhaskar S, Williams SG, Hillman KA, et al Pinpointing clinical diagnosis through whole exome sequencing to direct patient care: a case of Senior-Loken syndrome. The Lancet. 385: 1916 10.1016/S0140-6736(15)60496-2 PMC7614377 25987160

[47] Chilamakuri CSR, Lorenz S, Madoui M-A, Vodák D, Sun J, Hovig E, et al Performance comparison of four exome capture systems for deep sequencing. BMC Genomics. 2014;15: 449 10.1186/1471-2164-15-449 24912484 PMC4092227

[48] Treangen TJ, Salzberg SL. Repetitive DNA and next-generation sequencing: computational challenges and solutions. Nat Rev Genet. 2012;13: 36–46. 10.1038/nrg3117 22124482 PMC3324860

[49] Vervoort R, Lennon A, Bird AC, Tulloch B, Axton R, Miano MG, et al Mutational hot spot within a new RPGR exon in X-linked retinitis pigmentosa. Nat Genet. 2000;25: 462–466. 10.1038/78182 10932196

[50] Huang X-F, Wu J, Lv J-N, Zhang X, Jin Z-B. Identification of false-negative mutations missed by next-generation sequencing in retinitis pigmentosa patients: a complementary approach to clinical genetic diagnostic testing. Genet Med Off J Am Coll Med Genet. 2015; 10.1038/gim.2014.193 25569437

[51] den Hollander AI, Koenekoop RK, Yzer S, Lopez I, Arends ML, Voesenek KEJ, et al Mutations in the CEP290 (NPHP6) gene are a frequent cause of Leber congenital amaurosis. Am J Hum Genet. 2006;79: 556–561. 10.1086/507318 16909394 PMC1559533

[52] Konrad M, Saunier S, Heidet L, Silbermann F, Benessy F, Calado J, et al Large homozygous deletions of the 2q13 region are a major cause of juvenile nephronophthisis. Hum Mol Genet. 1996;5: 367–371. 8852662 10.1093/hmg/5.3.367

[53] Maugeri A, van Driel MA, van de Pol DJ, Klevering BJ, van Haren FJ, Tijmes N, et al The 2588G—>C mutation in the ABCR gene is a mild frequent founder mutation in the Western European population and allows the classification of ABCR mutations in patients with Stargardt disease. Am J Hum Genet. 1999;64: 1024–1035. 10090887 10.1086/302323 PMC1377826

[54] Al-Gazali L, Ali BR. Mutations of a country: a mutation review of single gene disorders in the United Arab Emirates (UAE). Hum Mutat. 2010;31: 505–520. 10.1002/humu.21232 20437613

