# Supplementary material for: Retinal Diseases Caused by Mutations in Genes Not Specifically Associated with the Clinical Diagnosis
Source: PLoS One. 2016 Oct 27;11(10):e0165405. doi: 10.1371/journal.pone.0165405 (PMC5082937; doi:10.1371/journal.pone.0165405)
Supplement: S1 Table — (DOCX) [file pone.0165405.s002.docx]

**S1 Table. Summary of cases in initial analysis and in this study**

| **Disease** | **Total cases** | **Solved by initial analysis** | **Solved in this study** | **Total solved cases** | **Total unsolved cases** |
| --- | --- | --- | --- | --- | --- |
| RP | 98 | 72 (73%) | 5 (5%) | 77 (78%) | 22 (22%) |
| LCA | 13 | 6 (46%) | 2 (15%) | 8 (62%) | 5 (38%) |
| FEVR | 12 | 3 (25%) | 2 (17%) | 5 (42%) | 7 (58%) |
| Total | 123 | 81 (66%) | 9 (7%) | 90 (73%) | 33 (27%) |

#66 RP, 19 LCA, and 4 FEVR genes were initially analyzed for 98 RP, 13 LCA, and 12 FEVR cases, respectively.
